# Automatic Detection of Secundum Atrial Septal Defect in Children Based on Color Doppler Echocardiographic Images Using Convolutional Neural Networks

**DOI:** 10.3389/fcvm.2022.834285

**Published:** 2022-04-06

**Authors:** Wenjing Hong, Qiuyang Sheng, Bin Dong, Lanping Wu, Lijun Chen, Leisheng Zhao, Yiqing Liu, Junxue Zhu, Yiman Liu, Yixin Xie, Yizhou Yu, Hansong Wang, Jiajun Yuan, Tong Ge, Liebin Zhao, Xiaoqing Liu, Yuqi Zhang

**Affiliations:** ^1^Department of Pediatric Cardiology, Shanghai Children’s Medical Center, School of Medicine, Shanghai Jiao Tong University, Shanghai, China; ^2^Deepwise Artificial Intelligence Laboratory, Beijing, China; ^3^Pediatric Artificial Intelligence Clinical Application and Research Center, Shanghai Children’s Medical Center, School of Medicine, Shanghai Jiao Tong University, Shanghai, China; ^4^Shanghai Engineering Research Center of Intelligence Pediatrics (SERCIP), Shanghai, China

**Keywords:** artificial intelligence, convolutional neural networks, automatic detection, secundum atrial septal defect, echocardiogram

## Abstract

Secundum atrial septal defect (ASD) is one of the most common congenital heart diseases (CHDs). This study aims to evaluate the feasibility and accuracy of automatic detection of ASD in children based on color Doppler echocardiographic images using convolutional neural networks. In this study, we propose a fully automatic detection system for ASD, which includes three stages. The first stage is used to identify four target echocardiographic views (that is, the subcostal view focusing on the atrium septum, the apical four-chamber view, the low parasternal four-chamber view, and the parasternal short-axis view). These four echocardiographic views are most useful for the diagnosis of ASD clinically. The second stage aims to segment the target cardiac structure and detect candidates for ASD. The third stage is to infer the final detection by utilizing the segmentation and detection results of the second stage. The proposed ASD detection system was developed and validated using a training set of 4,031 cases containing 370,057 echocardiographic images and an independent test set of 229 cases containing 203,619 images, of which 105 cases with ASD and 124 cases with intact atrial septum. Experimental results showed that the proposed ASD detection system achieved accuracy, recall, precision, specificity, and F1 score of 0.8833, 0.8545, 0.8577, 0.9136, and 0.8546, respectively on the image-level averages of the four most clinically useful echocardiographic views. The proposed system can automatically and accurately identify ASD, laying a good foundation for the subsequent artificial intelligence diagnosis of CHDs.

## Introduction

Congenital heart diseases (CHDs) are one of the most common congenital birth defects. The incidence of CHD is about 0.9% among the newborns born in China (1). Atrial septal defect (ASD) is considered to be one of the most common CHDs, and the estimated prevalence of ASD is 0.88 per 1,000 patients. The most common type of ASD is the secundum ASD, which accounts for approximately 80% of ASD (2). Echocardiography is noninvasive, nonradioactive, and can comprehensively evaluate the structure and function of the heart and blood vessels, and is widely used in the diagnosis and treatment of cardiovascular malformations. In China, because of the large pediatric population, there is a huge demand for echocardiography specialists for CHD diagnosis. Echocardiographic diagnosis relies on the operator to collect video streams from different perspectives and observe the morphology of organs and tissues from multiple views. Accurate diagnosis is largely affected by the operator’s personal technical skills. However, due to the long training time to be an echocardiography expert, it is difficult to diagnose CHDs accurately for most of primary hospitals lacking experienced echocardiography experts. Therefore, there is an urgent need to develop an automatic diagnostic system based on echocardiographic analysis that can quickly and accurately diagnose CHDs and assist echocardiography operators to reduce misdiagnosis caused by artificial factors.

In recent years, with the development of artificial intelligence (AI) technology, deep learning methods based on convolutional neural networks (CNNs) have been applied to various medical image analysis tasks, including lesion detection, organ segmentation, and disease diagnosis. Recent studies have shown that object detection technology can be used to detect lesions of knee joint (3), thyroid (4), breast (5), pancreas (6), and other diseases. However, there are very few reports on detection of abnormal cardiac structures. The U-Net based architecture has also been widely applied in many segmentation tasks, such as liver (7, 8), lung (9), tumor segmentation (10, 11), and prostate (12, 13). U-Net has also attracted many attentions in the field of ultrasonic images such as segmentation of ovary (14), fetal head (15), and breast (16). As for CHD diagnosis, it has also been reported that AI-based automatic auscultation may improve the accuracy of CHD screening (17). However, the application of automatic auscultation in the diagnosis of CHD is limited since it cannot accurately diagnose the type of CHDs, measure the size of the defect, and, further evaluate hemodynamics (such as shunt direction).

Advances in the digitization, standardization, and storage of echocardiograms have led to recent interest in the automatic interpretation of echocardiograms based on deep learning. Current research on echocardiographic analysis focused on detecting abnormal ventricular function and locating ventricular wall motion (18–20). Nevertheless, existing work of ventricular segmentation (21), cardiac phase detection (22), ejection fraction assessment (23), and other tasks still cannot meet the needs of accurate diagnosis of CHDs. Standard view recognition based on echocardiography is a prerequisite for clinical diagnosis of heart diseases. Baumgartner et al. (24) proposed a two-dimensional CNN containing six convolutional modules, which can recognize 12 standard views of fetal ultrasound with an average accuracy of 0.69 and an average recall rate of 0.80. Sridar et al. (25) used the pre-trained AlexNet to identify 14 views of fetuses and achieved a precision of 0.76 and a recall of 0.75 on average. Madani et al. (26) and Howard et al. (27) also trained CNN-based models to classify 15 standard echocardiographic views with reasonable results. However, these tasks used large networks with high computational complexity to achieve high performance and require high-standard hardware configurations, which may not meet the real-time requirements of CHD diagnosis in practice. Recent advances on CNNs have also led to rapid progress in multiple standard view recognition for echocardiography (26, 28, 29), with an overall accuracy of 97 or 98%. However, these works were for adults and may not be suitable for ASD detection in children.

In this study, we proposed an automatic ASD detection system which can perform image-level ASD detection based on color Doppler echocardiographic images using CNNs. The proposed automatic ASD detection system consists of four modules, namely the standard view identification module, the cardiac anatomy segmentation module, the ASD candidate detection module and the detection refinement module. In clinical diagnosis, due to the complexity of the heart structure and the limitations of two-dimensional echocardiographic scanning of ASDs, especially posterior inferior border defect detection, clinicians need to examine the heart from different views. In addition, some clinical signs can only be observed from certain views. We used multiple sites (subxiphoid, apical, and parasternal) and multiple views to simulate the diagnosis by sonographers in real clinical scenarios instead of using a single view. The standard view identification module is designed to identify four clinical meaningful echocardiographic views (that is, the subcostal view focusing on the atrium septum, the apical four-chamber view, the low parasternal four-chamber view, and the parasternal short-axis view) that are most useful for diagnosing ASD. The cardiac anatomy segmentation module aims to segment the left atrium (LA) and the right atrium (RA) from the images of the four target standard views, since ASD occurs in the septal area between LA and RA. The ASD candidate detection module finds all ASD candidates, and finally the detection refinement module applies deterministic spatial analysis to further refine the ASD detection results based on the information derived from the output of the cardiac anatomy segmentation. The proposed ASD detection system was developed and validated using a training set of 4,031 cases containing 370,057 echocardiographic images and an independent test set of 229 cases containing 203,619 images. Experimental results show the proposed system can automatically and accurately detect ASD, paving the way for the automatic diagnosis of CHD.

The main contributions of this study include: firstly, to our knowledge, a fully automatic CNN-based ASD detection system was proposed for the first time; secondly, we established a data set consists of a training set of 370,057 images of 4,031 cases and a test set of 203,619 images of 229 cases, that meets the requirements of ASD clinical diagnosis and is the largest dataset reported so far; thirdly, our standard view identification model has achieved the state of the art recognition performance with an the average accuracy of 0.9942 and F1 score of 0.9377 for the four target views of ASD diagnosis while using a small network through knowledge distillation which meet the real-time requirements of CHD diagnosis in practice; fourthly, the newly introduced dense dual attention mechanism in the cardiac anatomy segmentation can improve segmentation performance by simultaneously aggregating context and location information; and finally experimental results proved that the proposed detection refinement module can effectively improve the detection precision while keep the recall rate basically unchanged.

## Materials and Methods

### Participants

The subject of this retrospective study is color Doppler echocardiographic images of pediatric patients undergoing ASD examination at Shanghai Children’s Medical Center. The time period for these examinations is from September 2018 to April 2021. These cases include patients diagnosed as positive and negative. Among them, positive cases were diagnosed as ASD with a diameter greater than 5 mm, and negative cases were diagnosed as intact atrial septum.

### Data Collection

The study has been approved by the Institutional Review Board of Shanghai Children’s Medical Center (Approval No. SCMCIRB-W2021058) and a patient exemption has been applied for. All patients were examined with echocardiography using Philips iE33, EPIQ 7C, and GE Vivid E95 ultrasound systems with S5-1, S8-3, M5Sc, and 6S transducers. Standard imaging techniques were used for two-dimensional, M-mode, and Color Doppler echocardiography in accordance with the recommendations of the American Society of Echocardiography (30). All data used in this study were randomly selected cases from Shanghai Children’s Medical Center’s PACS database and these cases were collected by different doctors on different ultrasound machines. All data were strictly desensitized to protect patient privacy. The original data format of echocardiography was DICOM video stored in the PACS database. In order to facilitate program processing, DICOM video was divided frame-by-frame into a series of JPEG images. The human heart is not a static organ, it is constantly contracting and expanding. ASD size and shunts also vary with the cardiac cycle. Therefore, we dynamically sample and collect a series of image frames from different cardiac cycles. Five junior clinicians were recruited to manually label the data, including view types, outlines of cardiac structures, and ASD diagnostic annotations. Diagnosis was made by analyzing the heart using image segments from different views (subcostal-, apex-, parasternal-, and suprasternal views, etc.). All manually annotated data were further reviewed and confirmed as the gold standard by two senior clinicians. During systole, diastole, and torsion of the heartbeat, the position of the atrial septum changes to some extent. The atrial septum may be blurred (especially in the subxiphoid view) due to motion artifacts. In this study, images with motion blur were excluded after expert review.

#### Training/Validation Dataset

A total of 4,031 cases (370,057 images) were used as the training set of the standard view recognition module, the cardiac anatomy segmentation module and the ASD detection module. Since our training set is large enough to adequately represent the data distribution, we sample and collect image frames using fewer cardiac cycles for each case. The dataset was randomly divided into training and validation sets and selectively annotated as shown in Table 1. Since the data are collected from a real clinical practice, the view distribution is basically the same as the daily diagnosis.

**TABLE 1:** Summary of the training and validation data sets.

	Training set		Validation set	
	Number	Number	Number	Number
	of cases	of images	of cases	of images
Standard view identification	3,409	247,750	96	102,904
Cardiac anatomy segmentation	237	7,500	101	2,185
Atrial septal defect detection	150	8,355	38	1,363

#### Test Dataset

Additional 105 ASD patients (32 male, median age of 1.80 years) and 124 normal controls with intact atrial septum (45 male, median age of 2.09 years) were enrolled as an independent test data set for the final ASD detection evaluation (Table 2). In order to thoroughly test the performance of the model, we sampled and collected image frames with more cardiac cycles for each case. As a result, a total of 203,619 echocardiography images were included (92,616 images in the ASD group and 111,003 images in the normal group). Table 2 shows the view distribution as well as clinical characteristics of the two groups of the test dataset. According to the recognition results of the standard view identification module, the data of the four target standard views were used to evaluate the performance of ASD detection. As shown in Table 2, there are a total of 40,264 images, including 18,338 images from ASD patients and 21,926 images from a normal control groups.

**TABLE 2:** Clinical characteristic and view distribution comparisons between the ASD group and the normal group of the test data set.

	ASD group (*n* = 105)	Normal group (*n* = 124)	*p*-Value/total
Age (years)	1.80 (0.04–14.46)	2.09 (0.11–14.61)	*p* = 0.25
Female/male	73/32	79/45	*p* = 0.40
Weight (kg)	11.00 (3.45–52.00)	12.50 (4.30–50.00)	*p* = 0.082
Height (cm)	80.00 (45.00–162.00)	90.00 (51.00–152.00)	*p* = 0.073
Size of ASD (mm)	12.1 ± 5.2	/	
Associated cardiac conditions	PDA (*n* = 2)	Small PDA (*n* = 3)	
	VSD (*n* = 11)	VSD (*n* = 6)	
	PS (*n* = 4)	Post PDA occlusion (*n* = 4)	
subAS (ASD detection/total) (*n*)	7,503/8,079	8,498/8,790	16,001/16,869
A4C (ASD detection/total) (*n*)	2,942/3,078	3,840/4,003	6,782/7,081
LPS4C (ASD detection/total) (*n*)	4,410/4,798	4,714/5,159	9,124/9,957
Sax-basal (ASD detection/total) (*n*)	3,483/4,245	4,874/5,689	8,357/9,934
Other (*n*)	72,416	87,362	159,778
Total (*n*)	92,616	111,003	203,619

*ASD, atrial septal defect; VSD, ventricular septal defect; PDA, patent ductus arteriosus; PS, pulmonary stenosis; subAS, subcostal atrium septum; A4C, apical four-chamber; LPS4C, low parasternal four-chamber.*

### Proposed Method

**FIGURE 1:**
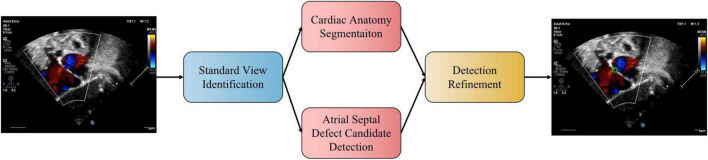
The pipeline of the proposed automatic ASD detection system. In practice, the two stages of cardiac anatomy segmentation and atrial septal defect candidate detection can be run in parallel.

We propose a three-stage ASD detection system, which includes four modules, namely, standard view identification, cardiac anatomy segmentation, ASD detection, and detection refinement, as shown in Figure 1. The first stage is the identification of standard view module, aiming to extract four target standard views, namely, the subcostal view focusing on the atrium septum (subAS), the apical four-chamber view (A4C), the low parasternal four-chamber view (LPS4C), and the sax-basal view, from frames of dynamic videos. The second stage includes cardiac anatomy segmentation and ASD detection modules. The former is to segment the target cardiac anatomy, and the latter is to detect candidate ASDs. The input of these two modules is the image of the target standard view extracted in the first stage. The third stage is the detection refinement module, which combines the results of the second stage to obtain refined detection results.

#### Standard View Identification

**FIGURE 2:**
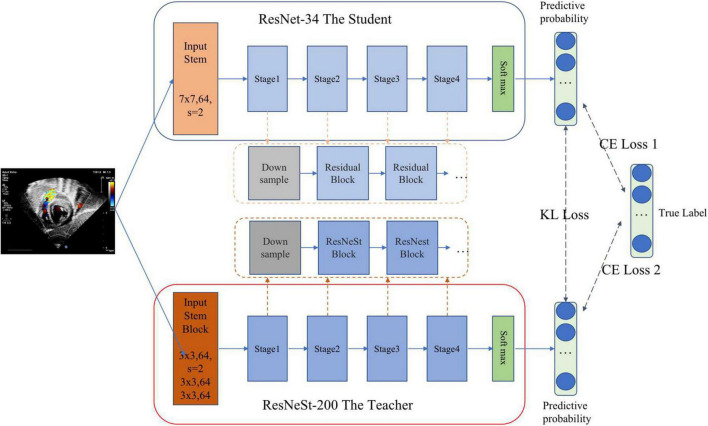
Standard view identification through knowledge distillation. The pre-training teacher–student based on KL (Kullback–Leibler) loss realizes knowledge transfer through joint training. Then the student is fine-tuned on training data based on CE (cross entropy) loss to complete the training process of knowledge distillation.

Standard echocardiographic view recognition is a prerequisite for clinical diagnosis of heart disease. Our standard view identification model is based on our previous work (31), where we recognized 24 classes of standard views with high accuracy. Since the purpose of this study is to detect ASD, we only focus on four target views (i.e., subAS, A4C, LPS4C, and sax-basal) and refer to all other views as “other.” As shown in Figure 2, a knowledge distillation (32) method was applied to train the standard view identification model, in which we applied ResNet-34 (33) as the student model and ResNeSt-200 (34) as the teacher model. We first trained a ResNeSt-200 network with a large amount of parameters, and then transferred the “knowledge” to a ResNet-34 network with a small amount of parameters through knowledge distillation. By minimizing the Kullback–Leibler divergence between the probability distributions of teacher and student models, knowledge transfer was achieved through joint training. During the training process, data augmentation methods were also applied, including horizontal random flip, vertical random flip, and polar coordinate rotation. It needs to be noted that the teacher model was only used during the training phase, and the small student model ResNet34 was used for inference.

#### Cardiac Anatomy Segmentation

**FIGURE 3:**
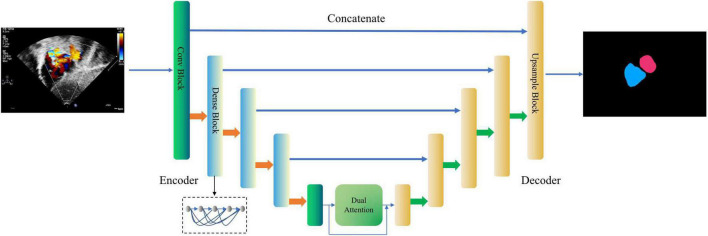
Dense Dual Attention U-Net based segmentor for cardiac anatomy segmentation. The second, third, and fourth layers of the encoder apply the dense blocks, containing 2, 4, and 8 dense layers, respectively, with a growth rate of 32. The dual attention module includes a position attention module and a channel attention module, respectively. The two modules process the input in parallel, and the two outputs are fused by addition.

This module is designed to segment the LA and the RA from the images of the four target standard views. As shown in Figure 3, a new encoder-decoder network called Dense Dual Attention U-Net is proposed as the atrium segmentor. The encoder gradually extracts features from the input image to obtain a high-dimensional representation of the image. The decoder reconstructs the image according to the high dimensional feature representation, and then outputs the segmentation mask. Jumping out of the tradition of U-Net (35), the hierarchical output features of the encoder are input to the decoder one by one through the “skip connection” mechanism for feature fusion. The convolutional layers of Dense Dual Attention U-Net adopts “dense connection” (36), and the encoder also uses “dual attention” (37), which are spatial-based and channel-based attentions, respectively. The dense dual attention mechanism introduced in the U-Net architecture can improve segmentation performance by simultaneously aggregating context and location information.

#### Atrial Septal Defect Candidate Detection

**FIGURE 4:**
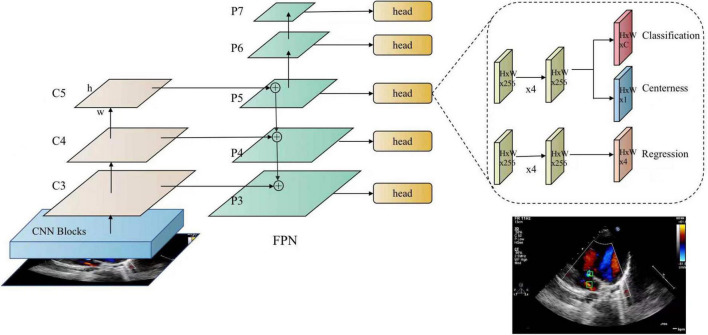
FCOS detector for atrial septal defects. FCOS consists of three parts, including the backbone (CNN), neck (FPN), task-special heads (classification, centerness, and regression).

This module aims to detect ASD candidates from the images of the four target standard views and mark the detected ASD candidates with confidence values. In this study, a fully convolutional single-stage object detector, known as FCOS (38) is applied as the ASD detector. As shown in Figure 4, FCOS has two output heads. The classification head outputs the class probability of the detected ASD candidate, i.e., the confidence of the detected ASD candidate, and the regression head outputs the coordinates of the candidate ASD area. The size of ASD varies greatly. The detection of large ASD relies on a large receptive field while the detection of small ASD relies on a high-resolution feature map. The feature pyramid network (FPN) (39) module in FCOS can handle this problem. In addition, FCOS has achieved a good balance between detection accuracy and computational complexity, meeting the real-time requirements of the proposed system.

#### Detection Refinement

**FIGURE 5:**
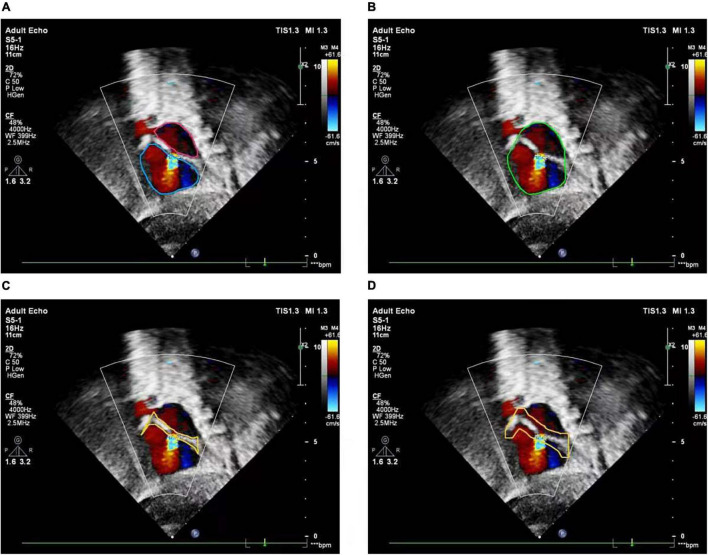
Atrial septal region extraction. **(A)** Segmented left and right atria, **(B)** convex hull embracing segmented left and right atria, **(C)** region differences between **(A)** and **(B)**, **(D)** morphologically dilated atrial septum.

**FIGURE 6:**
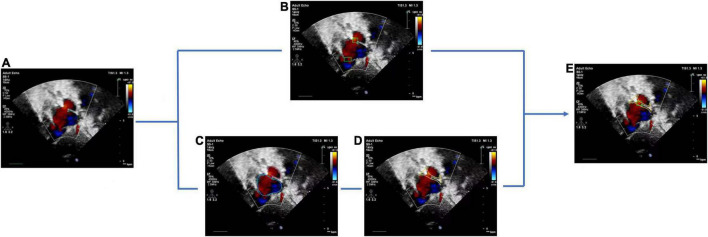
Atrial septal defect detection refinement. **(A)** Input image with a target view, **(B)** detected ASD candidates, **(C)** segmentation result of cardiac anatomy, **(D)** extraction of atrial septal region based on **(C)**, **(E)** final refined result of ASD detection.

The basic rule of ASD diagnosis is that ASD occurs in the septum area between LA and RA. Theoretically, the ASD candidates detected by the FCOS detector may appear in any area of the image. Therefore, detection refinement is necessary to filtered out false positives detected. Based on the outputs of the cardiac anatomy segmentation, the septum area can be extracted through deterministic spatial analysis. More specifically, we first need to find the smallest convex hull of LA and RA, and then the difference between the convex hull and the area of LA and RA is the septum area. As shown in Figure 5, considering the decision margin, morphological dilation techniques can be used to expand the septum area. Finally, ASD candidates detected outside the septum area are regarded as false positives and filtered, as shown in Figure 6.

#### Environment Configuration

All codes were implemented using Python 3.7 and Pytorch 1.4.0. The experiment was conducted on a workstation platform with 8 NVIDIA TITANRTX GPUs, 24 GB GPU memory, 256 G RAM, and 80 Intel(R) Xeon(R) Gold 6248 CPU @ 2.50 GHz, using Ubuntu 16.04.

## Results

### Performance Evaluation

We use Accuracy, *Recall*, *Precision*, *Specificity*, and *F1 Score* to evaluate the performance of view identification and ASD detection and apply *Dice Similarity Coefficient (DSC)* as the performance evaluation metric for cardiac anatomical segmentation. They are defined as follows:


(1) $$A\,c\,c\,u\,r\,a\,c\,y=\frac{T\,P+T\,N}{T\,P+F\,P+F\,N+T\,N}$$

(2) $$R\,e\,c\,a\,l\,l=S\,e\,n\,s\,i\,t\,i\,v\,i\,t\,y=\frac{T\,P}{T\,P+F\,N}$$

(3) $$P\,r\,e\,c\,i\,s\,i\,o\,n=\frac{T\,P}{T\,P+F\,P}$$

(4) $$S\,p\,e\,c\,i\,f\,i\,c\,i\,t\,y=\frac{T\,N}{T\,N+F\,P}$$

(5) $$F\,1\,S\,c\,o\,r\,e=2\times \frac{P\,r\,e\,c\,i\,s\,i\,o\,n\times R\,e\,c\,a\,l\,l}{P\,r\,e\,c\,i\,s\,i\,o\,n+R\,e\,c\,a\,l\,l}$$

(6) $$D\,S\,C=\frac{2\times \left|A\cap B\right|}{\left|A\right|+\left|B\right|}$$

Among them, TP, FP, TN, and FN are the counts of true positive, false positive, true negative, and false negative, respectively. TP and TN represent the positives and negatives of correct predictions with respect to the ground truth. FP and FN represent positives and negatives of incorrect predictions with respect to the ground truth. F1 score is the harmonic average of Precision and Recall with a value ranged in (0–1). The higher value, the better the model performance. A is defined as the ground truth area, and B is defined as the segmented area.

The receiver operating characteristic (ROC) curve is plotted by using *1-Specificity* as the *X*-axis and *Sensitivity* as the *Y*-axis. The area under the curve (AUC) is calculated based on the trapezoidal method to measure the detection performance. The best confidence cut-off point is determined according to the *Youden Index* defined as follows:


(7) Y⁢o⁢u⁢d⁢e⁢n⁢I⁢n⁢d⁢e⁢x=S⁢e⁢n⁢s⁢i⁢t⁢i⁢v⁢i⁢t⁢y+S⁢p⁢e⁢c⁢i⁢f⁢i⁢c⁢i⁢t-1

### Performance of Standard View Identification

The performance of the standard view recognition was evaluated on 203,619 echocardiographic images in the test data set. As shown in Table 3, our standard view identification model achieved excellent performance. For the four target views (i.e., subAS, A4C, LPS4C, and PSAX), the averages of accuracy, recall, precision, specificity, and F1 score were 0.9942, 0.9126, 0.9663, 0.9983, and 0.9377, respectively. The parameters of our model are about 21.3 M, which is less than 1/3 of the parameters of the teacher ResNeSt-200 model (approximately 70.2 M). In terms of computational complexity, the FLOPs of our model is about 3.7 G, which is only 1/5 of the FLOPs of ResNeSt-200 (about 13.48 G). Through knowledge distillation, it significantly reduced the computational cost while maintained the precision of network classification.

**TABLE 3:** Performance results of standard view identification.

View	Accuracy	Recall	Precision	Specificity	F1 score
subAS (95% CI)	0.9965 (0.9962–0.9969)	0.9485 (0.9452–0.9519)	0.9985 (0.9979–0.9991)	0.9999 (0.9998–0.9999)	0.9729 (0.9729–0.9729)
A4C (95% CI)	0.9975 (0.9972–0.9978)	0.9444 (0.9391–0.9497)	0.9788 (0.9754–0.9822)	0.9993 (0.9991–0.9994)	0.9613 (0.9613–0.9613)
LPS4C (95% CI)	0.9908 (0.9902–0.9914)	0.9163 (0.9109–0.9218)	0.8971 (0.8912–0.9031)	0.9946 (0.9943–0.9949)	0.9066 (0.9066–0.9067)
Sax-basal (95% CI)	0.9919 (0.9913–0.9924)	0.8413 (0.8341–0.8484)	0.9908 (0.9887–0.9928)	0.9996 (0.9995–0.9997)	0.9099 (0.9099–0.9099)
Mean	0.9942	0.9126	0.9663	0.9983	0.9377

*subAS, subcostal atrium septum; A4C, apical four-chamber; LPS4C, low parasternal four-chamber.*

### Performance of Cardiac Anatomy Segmentation

**FIGURE 7:**
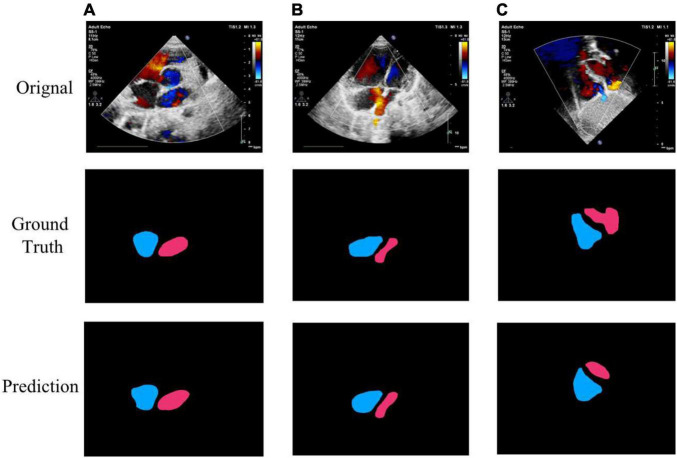
Example segmentation results of cardiac anatomy. **(A)** High precision segmentation of DSC 0.9517; **(B)** medium precision segmentation of DSC 0.9115; **(C)** poor segmentation performance of DSC 0.7347.

The performance of the cardiac anatomical segmentation was evaluated on 101 cases with 2,185 echocardiographic images in the validation data set. In this study, we categorized the verification data into four groups. More specifically, data containing only LA and RA was considered as A2C; data containing LA, RA, left ventricle, and right ventricle was regarded as A2C-V2C; data containing atrium and left ventricle was classified as A2C-LV; and data containing LA, RA, and right ventricle was categorized as A2C-RV. The number distribution of each group and the corresponding segmentation results were shown in Table 4, where we only focused on the segmentation results of the LA and the RA. Table 5 also demonstrated the ablation experimental results of the proposed Dense Dual Attention U-Net, which incorporated two additional modules, namely the Dense block and the Dual Attention modules. As shown in Table 5, both the Dense block and the Dual Attention had positive impacts on the segmentation performance of the U-Net. Figure 7 also demonstrated some of the example results of cardiac anatomical segmentation with high, medium and low performance.

**TABLE 4:** Performance results of cardiac anatomy segmentation.

	Number of images	Left atrium (DSC)	Right atrium (DSC)	Mean (DSC)
A2C	993	0.8960	0.9089	0.9025
A2C-V2C	731	0.8987	0.9239	0.9113
A2C-LV	31	0.8908	0.8816	0.8862
A2C-RV	430	0.8638	0.9171	0.8905
Total/mean	2,185	0.8873	0.9079	0.8976

*A2C, image containing left and right atria only; A2C-V2C, image containing left and right atria and left and right ventricles; A2C-LV, image containing left and right atria and only left ventricle; A2C-RV, image containing left and right atria and only right ventricle.*

**TABLE 5:** Performance of U-Net with different modules.

	U-Net	U-Net w/dense block	U-Net w/dual attention	Dense dual attention U-Net
A2C	0.8860	0.8969	0.8973	**0.9025**
A2C-V2C	0.8889	0.9044	0.9018	**0.9113**
A2C-LV	0.8814	0.8837	0.8716	**0.8862**
A2C-RV	0.8548	**0.8932**	0.8705	0.8905
Mean	0.8778	0.8945	0.8853	**0.8976**

*A2C, image containing left and right atria only; A2C-V2C, image containing left and right atria and left and right ventricles; A2C-LV, image containing left and right atria and only left ventricle; A2C-RV, image containing left and right atria and only right ventricle. Numbers in bold font indicate better performance in each category.*

### Performance of Atrial Septal Defect Detection

**FIGURE 8:**
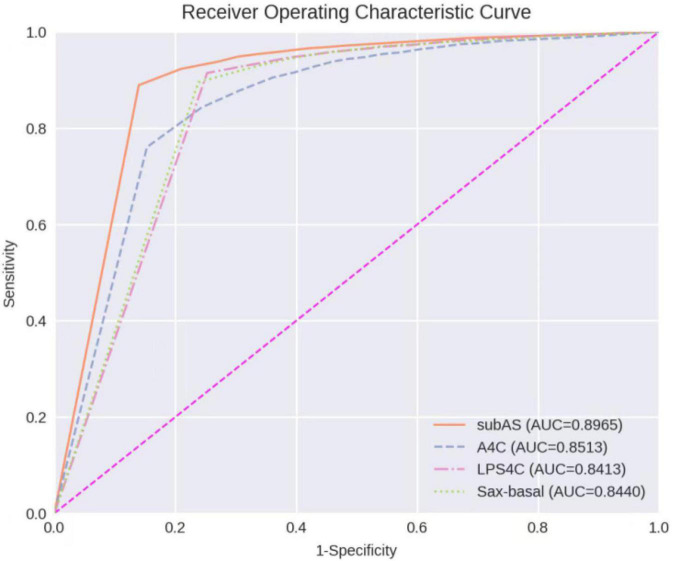
Receiver operating characteristic curves of ASD detection on four target echocardiographic views.

**FIGURE 9:**
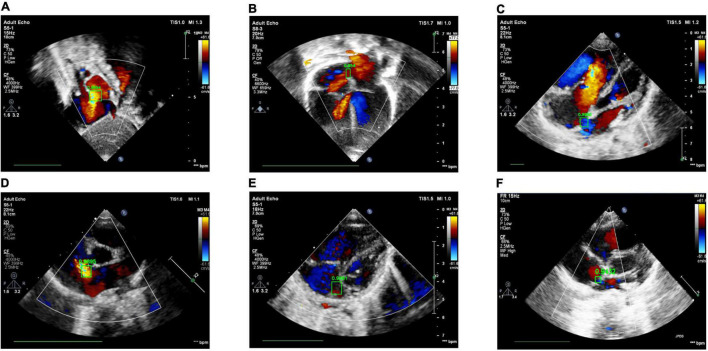
Examples of success and failure cases. **(A)** ASD detected in the subAS view: bright red shows the transeptal flow with left-to-right shunt, **(B)** ASD detected in the A4C view: dark red in the center of the atrial septum indicates the occurrence of left-to-right shunt flow, **(C)** ASD detected in the LPS4C view: blue regions represent the transeptal flow with right-to-left shunt, **(D)** ASD detected in the PSAX view: bright red shows the transeptal flow with left-to-right shunt. **(E)** ASD detection of false positive, due to the confusion of similar structures and the failure of the cardiac anatomy segmentation stage; **(F)** ASD detection of true negative, due to the low confidence (0.9432 < 0.95).

The ROC Curve of the ASD detection model on the four target echocardiographic views was illustrated in Figure 8. The AUC of subAS was the highest, reaching 0.8965, and the AUCs of the other three views were roughly at the same level, indicating that the model had a stronger ASD detection ability in the view of subAS. In this study, the optimal cut-point was determined by calculating the maximum value of *Youden Ind*ex. According to the analysis of AUC curves, the optimal cut-point was 0.95. Therefore, cases that are not detected or have a confidence level lower than 0.95 were considered as negatives and cases with a confidence level greater than or equal to 0.95 were regarded as positives. Figure 9 also showed example successful and failure cases of ASD detection. The ASD detection performances of before and after the detection refinement were compared in Table 6. The average values of *Accuracy*, *Recall*, *Precision*, *Specificity*, and *F1 Score* for image-level ASD detection before the detection refinement were 0.8699, 0.8608, 0.8208, 0.8744, and 0.8397, respectively, while the average values of *Accuracy*, *Recall*, *Precision*, *Specificity*, and *F1 Score* for image-level ASD detection after the detection refinement were 0.8833, 0.8545, 0.8577, 0.9136, and 0.8546, respectively. It can be seen that *Accuracy*, *Precision*, *Specificity*, and *F1 Score* have increased by 1.34, 3.69, 3.92, and 1.49%, respectively, while the recall rate has been reduced by only 0.63%. The *p*-values of the *t*-test indicated statistically significant differences in ASD detection before and after the refinement module for all performance metrics in all other views except the LPS4C view. As for view LPS4C, the differences in ASD detection before and after the refinement module were statistically significant in terms of recall, precision, and specificity, but not in terms of accuracy and F1 score. In addition, a preliminary case-level study has also been conducted where a threshold of 0.6 was used based on a prior from experienced physicians. As shown in Table 6, the average values of Accuracy, Recall, Precision, Specificity, and F1 Score for case-level ASD detection before the detection refinement were 0.9888, 0.8381, 0.8786, 0.9214, and 0.9072, respectively, while the average values of Accuracy, Recall, Precision, Specificity, and F1 Score for case-level ASD detection after the detection refinement were 0.9897, 0.9143, 0.9318, 0.9563, and 0.9505, respectively. A thorough grid-search based approach can be performed to find the optimal threshold in future studies when larger test sets are available.

**TABLE 6:** Performance results of ASD detection before vs after refinement.

View	Accuracy	Recall	Precision	Specificity	F1 score
subAS (95% CI)	0.8958 (0.8912–0.9005)/**0.9030**	**0.8755** (0.8678–0.8832)/0.8742	0.8771 (0.8694–0.8848)/**0.8909**	0.9106 (0.9049–0.9163)/**0.9329**	0.8763 (0.8762–0.8764)/**0.8825**
	(0.8985–0.9075) (*p* < 0.0001)	(0.8656–0.8828) (*p* < 0.0001)	(0.8827–0.8991) (*p* < 0.0001)	(0.9277–0.9381) (*p* < 0.0001)	(0.8824–0.8825) (*p* < 0.0001)
A4C (95% CI)	0.8220 (0.8132–0.8309)/**0.8500**	**0.7540** (0.7375–0.7705)/0.7352	0.7601 (0.7437–0.7765)/**0.8461**	0.8616 (0.8515–0.8717)/**0.9388**	0.7570 (0.7568–0.7572)/**0.7868**
	(0.8418–0.8583) (*p* < 0.0001)	(0.7153–0.7552) (*p* < 0.0001)	(0.8286–0.8636) (*p* = 0.00)	(0.9314–0.9461) (*p* = 0.00)	(0.7866–0.7870) (*p* < 0.0001)
LPS4C (95% CI)	0.8860 (0.8798–0.8922)/**0.8990**	0.9080 (0.8995–0.9166)/0.9080	0.8447 (0.8344–0.8550)/**0.8734**	0.8687 (0.8598–0.8775)/**0.9020**	0.8752 (0.8751–0.8753)/**0.8904**
	(0.8931–0.9049) (*p* = 0.38)	(0.8984–0.9176) (*p* < 0.0001)	(0.8626–0.8842) (*p* < 0.0001)	(0.8935–0.9106) (*p* < 0.0001)	(0.8902–0.8905) (*p* = 0.62)
Sax-basal (95% CI)	0.8758 (0.8693–0.8824)/**0.8813**	**0.9058** (0.8965–0.9151)/0.9005	0.8013 (0.7894–0.8132)/**0.8203**	0.8567 (0.8478–0.8656)/**0.8808**	0.8504 (0.8502–0.8505)/**0.8585**
	(0.8750–0.8877) (*p* < 0.0001)	(0.8897–0.9113) (*p* = 0.0016)	(0.8071–0.8335) (*p* < 0.0001)	(0.8717–0.8898) (*p* < 0.0001)	(0.8584–0.8587) (*p* < 0.0001)
Mean	0.8699/**0.8833**	**0.8608**/0.8545	0.8208/**0.8577**	0.8744/**0.9136**	0.8397/**0.8546**
Case-level	0.9888/**0.9897**	0.8381/**0.9143**	0.8786/**0.9318**	0.9214/**0.9563**	0.9072/**0.9505**

*subAS, subcostal atrium septum; A4C, apical four-chamber; LPS4C, low parasternal four-chamber. A p-value (p < 0.05) indicates statistically significant. Numbers in bold font indicate better performance in each category.*

## Discussion

In this study, we proposed a CNN-based ASD detection system, which consists of three stages. In the first stage, four target standard views are extracted from the echocardiographic video frames. In the second stage, the cardiac anatomy and ASD candidates are obtained, separately. Finally, the third stage combines the two results of the second stage to refine and obtain the final ASD detection result. In practice, the cardiac anatomy segmentation and ASD candidate detection in the second stage can be run in parallel to meet the real-time requirements of CHD diagnosis. In our study, the floating point operations per second (FLOPs) of the ResNet-34 standard view identification module, the Dense Dual Attention U-Net, and the FCOS ASD detector were about 3.7 G, 130.28, and 219.25 G, respectively.

The proposed ASD detection system was developed using a training set of 4,031 cases containing 370,057 echocardiograms. The experimental results on an independent test set of 229 cases showed that the proposed system can accurately identify ASD in color Doppler echocardiographic images, which provides a good preparation for subsequent AI-based CHD diagnosis. Ideally, we should conduct additional ablation studies on the impact of each module on the final ASD detection. However, currently, due to the huge cost of data labeling, currently, our independent test data only has ASD labels for each image without segmentation ground truth. Therefore, we take this as one of the limitations and future work. As for the standard view identification module, since the overall accuracy of 0.9942 is high enough, the impact of failure cases of this module should be negligible.

Based on our clinical experience, small defects may close spontaneously in childhood, while large defects may cause hemodynamic abnormalities and clinical symptoms if they are not repaired in time. In addition, the hemodynamics of long-term left-to-right shunt significantly increase the possibility of late clinical complications, including functional decline, atrial arrhythmia, and pulmonary hypertension. Therefore, in this study, we have selected cases with a defect size of more than 5 mm as our research object. Theses cases may have abnormal hemodynamics and require surgery or transcatheter closure.

The *F1 Score* of ASD detection for images of the A4C view is relatively low compared to images of the other three views. Atrial septum is a relatively thin structure, especially in the fossa ovalis area. According to clinical expertise, subcostal, and the parasternal views are particularly useful for ASD diagnosis, because in these views, the septum is aligned almost perpendicular to the ultrasound beam. The thin area of the atrial septum and the color shunt flow can be particularly well resolved in these views. On the other hand, because the atrial septum is aligned parallel to the ultrasound beam in the A4C view, it is challenging to diagnose ASD with certainty in this view. Therefore, our experimental results are consistent with clinical practice.

Our model was trained and tested based on the Asian children. Although there is no literature evidence for differences in ASD by ethnicity, we may evaluate our model performance of different ethnic groups as one of the possible future studies. The acoustic window degenerates with age, especially in the subcostal view. It is not clear whether the proposed method can detect small ASD in adults, which will be further explored in future studies.

In our study, we found that ASD shunt blood flow was not present in every frame of the cardiac cycle due to the contraction, relaxation and torsion of the heartbeat. Image-level detection is the basis for case-level diagnosis. This research was the first attempt to identify ASD in children at the image level. A preliminary case-level study has also been conducted where a threshold of 0.6 was used based on a prior from experienced physicians. A thorough grid-search based approach can be performed to find the optimal threshold value in future studies when larger test sets are available. In addition, future research may also be to discover hidden patterns embedded in the cardiac cycle and to design case-level diagnostic models for ASD. It is well known that echocardiography cannot avoid the influence of color noise and the system performance largely depends on the quality of the original images. How to integrate the proposed system into the actual clinical diagnosis of ASD will be another direction of future research.

## Data Availability Statement

The raw data supporting the conclusions of this article will be made available for research purposes only upon request.

## Ethics Statement

Study approval was granted by the Institutional Review Board of Shanghai Children’s Medical Center (SCMCIRB-W2021058). The procedures were performed in accordance with the Declaration of Helsinki and International Ethical Guidelines for Biomedical Research Involving Human Subjects. Written informed consent to participate in this study was provided by the participants’ legal guardian/next of kin.

## Author Contributions

WH, YZ, XL, and YY: conception and design. YZ, YY, and LiZ: administrative support. WH, LW, and LC: provision of study materials or patients, and collection and assembly of data. QS, XL, WH, and YY: data analysis and interpretation. All authors wrote and final approval of manuscript.

## Conflict of Interest

The authors declare that the research was conducted in the absence of any commercial or financial relationships that could be construed as a potential conflict of interest.

## Publisher’s Note

All claims expressed in this article are solely those of the authors and do not necessarily represent those of their affiliated organizations, or those of the publisher, the editors and the reviewers. Any product that may be evaluated in this article, or claim that may be made by its manufacturer, is not guaranteed or endorsed by the publisher.

## References

[1] Zhao QM Liu F Wu L Ma XJ Niu C Huang GY. Prevalence of congenital heart disease at live birth in China. *J Pediatr.* (2019) 204:53–8. 10.1016/j.jpeds.2018.08.040 30270157

[2] Bradley EA Zaidi AN. Atrial septal defect. *Cardiol Clin.* (2020) 38:317–24. 10.1016/j.ccl.2020.04.001 32622487

[3] Liu F Zhou Z Samsonov A Blankenbaker D Larison W Kanarek A Deep learning approach for evaluating knee MR images: achieving high diagnostic performance for cartilage lesion detection. *Radiology.* (2018) 289:160–9. 10.1148/radiol.2018172986 30063195 PMC6166867

[4] Ma J Wu F Jiang T Zhu J Kong D. Cascade convolutional neural networks for automatic detection of thyroid nodules in ultrasound images. *Med Phys.* (2017) 44:1678–91. 10.1002/mp.12134 28186630

[5] Cao Z Duan L Yang G Yue T Chen Q. An experimental study on breast lesion detection and classification from ultrasound images using deep learning architectures. *BMC Med Imaging.* (2019) 19:51. 10.1186/s12880-019-0349-x 31262255 PMC6604293

[6] Chu LC Park S Kawamoto S Wang Y Zhou Y Shen W Application of deep learning to pancreatic cancer detection: lessons learned from our initial experience. *J Am Coll Radiol.* (2019) 16(9 Pt B):1338–42. 10.1016/j.jacr.2019.05.034 31492412

[7] Seo H Huang C Bassenne M Xiao R Xing L. Modified U-net (mU-Net) with incorporation of object-dependent high level features for improved liver and liver-tumor segmentation in CT images. *IEEE Trans Med Imaging.* (2020) 39:1316–25. 10.1109/TMI.2019.2948320 31634827 PMC8095064

[8] Jin Q Meng Z Sun C Cui H Su R. RA-UNet: a hybrid deep attention-aware network to extract liver and tumor in CT scans. *Front Bioeng Biotechnol.* (2020) 23:605132. 10.3389/fbioe.2020.605132 33425871 PMC7785874

[9] Yahyatabar M Jouvet P Cheriet F. Dense-Unet: a light model for lung fields segmentation in chest X-ray images. *Annu Int Conf IEEE Eng Med Biol Soc.* (2020) 2020:1242–5. 10.1109/EMBC44109.2020.9176033 33018212

[10] Meng Z Fan Z Zhao Z Su F. ENS-Unet: end-to-end noise suppression U-net for brain tumor segmentation. *Annu Int Conf IEEE Eng Med Biol Soc.* (2018) 2018:5886–9. 10.1109/EMBC.2018.8513676 30441675

[11] Yang T Zhou Y Li L Zhu C. DCU-net: multi-scale U-net for brain tumor segmentation. *J Xray Sci Technol.* (2020) 28:709–26. 10.3233/XST-200650 32444591

[12] Aldoj N Biavati F Michallek F Stober S Dewey M. Automatic prostate and prostate zones segmentation of magnetic resonance images using denseNet-like U-net. *Sci Rep.* (2020) 10:14315. 10.1038/s41598-020-71080-0 32868836 PMC7459118

[13] Machireddy A Meermeier N Coakley F Song X. Malignancy detection in prostate multi-parametric MR images using U-net with attention. *Annu Int Conf IEEE Eng Med Biol Soc.* (2020) 2020:1520–3. 10.1109/EMBC44109.2020.9176050 33018280

[14] Li H Fang J Liu S Liang X Yang X Mai Z CR-Unet: a composite network for ovary and follicle segmentation in ultrasound images. *IEEE J Biomed Health Inform.* (2020) 24:974–83. 10.1109/JBHI.2019.2946092 31603808

[15] Ashkani Chenarlogh V Ghelich Oghli M Shabanzadeh A Sirjani N Akhavan A Shiri I Fast and accurate U-net model for fetal ultrasound image segmentation. *Ultrason Imaging.* (2022) 6:1617346211069882. 10.1177/01617346211069882 34986724

[16] Amiri M Brooks R Behboodi B Rivaz H. Two-stage ultrasound image segmentation using U-net and test time augmentation. *Int J Comput Assist Radiol Surg.* (2020) 15:981–8. 10.1007/s11548-020-02158-3 32350786

[17] Thompson WR Reinisch AJ Unterberger MJ Schriefl AJ. Artificial intelligence-assisted auscultation of heart murmurs: validation by virtual clinical trial. *Pediatr Cardiol.* (2019) 40:623–9. 10.1007/s00246-018-2036-z 30542919

[18] Sudarshan V Acharya UR Ng EY Meng CS Tan RS Ghista DN. Automated identification of infarcted myocardium tissue characterization using ultrasound images: a review. *IEEE Rev Biomed Eng.* (2015) 8:86–97. 10.1109/RBME.2014.2319854 24803322

[19] Kusunose K Abe T Haga A Fukuda D Yamada H Harada M A deep learning approach for assessment of regional wall motion abnormality from echocardiographic images. *JACC Cardiovasc Imaging.* (2020) 13(2 Pt 1):374–81. 10.1016/j.jcmg.2019.02.024 31103590

[20] Kusunose K Haga A Yamaguchi N Abe T Fukuda D Yamada H Deep learning for assessment of left ventricular ejection fraction from echocardiographic images. *J Am Soc Echocardiogr.* (2020) 33:632–5.e1. 10.1016/j.echo.2020.01.009 32111541

[21] Yu L Guo Y Wang Y Yu J Chen P. Segmentation of fetal left ventricle in echocardiographic sequences based on dynamic convolutional neural networks. *IEEE Trans Biomed Eng.* (2017) 64:1886–95. 10.1109/TBME.2016.2628401 28113289

[22] Taheri Dezaki F Liao Z Luong C Girgis H Dhungel N Abdi AH Cardiac phase detection in echocardiograms with densely gated recurrent neural networks and global extrema loss. *IEEE Trans Med Imaging.* (2019) 38:1821–32. 10.1109/TMI.2018.2888807 30582532

[23] Jafari MH Girgis H Van Woudenberg N Liao Z Rohling R Gin K Automatic biplane left ventricular ejection fraction estimation with mobile point-of-care ultrasound using multi-task learning and adversarial training. *Int J Comput Assist Radiol Surg.* (2019) 14:1027–37. 10.1007/s11548-019-01954-w 30941679

[24] Baumgartner CF Kamnitsas K Matthew J Fletcher TP Smith S Koch LM SonoNet: real-time detection and localisation of fetal standard scan planes in freehand ultrasound. *IEEE Trans Med Imaging.* (2017) 36:2204–15. 10.1109/TMI.2017.2712367 28708546 PMC6051487

[25] Sridar P Kumar A Quinton A Nanan R Kim J Krishnakumar R. Decision fusion-based fetal ultrasound image plane classification using convolutional neural networks. *Ultrasound Med Biol.* (2019) 45:1259–73. 10.1016/j.ultrasmedbio.2018.11.016 30826153

[26] Madani A Arnaout R Mofrad M Arnaout R. Fast and accurate view classification of echocardiograms using deep learning. *NPJ Digit Med.* (2018) 1:6. 10.1038/s41746-017-0013-1 30828647 PMC6395045

[27] Howard JP Tan J Shun-Shin MJ Mahdi D Nowbar AN Arnold AD Improving ultrasound video classification: an evaluation of novel deep learning methods in echocardiography. *J Med Artif Intell.* (2020) 25:4. 10.21037/jmai.2019.10.03 32226937 PMC7100611

[28] Østvik A Smistad E Aase SA Haugen BO Lovstakken L. Real-time standard view classification in transthoracic echocardiography using convolutional neural networks. *Ultrasound Med Biol.* (2019) 45:374–84. 10.1016/j.ultrasmedbio.2018.07.024 30470606

[29] Zhang J Gajjala S Agrawal P Tison GH Hallock LA Beussink N Fully automated echocardiogram interpretation in clinical practice. *Circulation.* (2018) 138:1623–35. 10.1161/CIRCULATIONAHA.118.034338 30354459 PMC6200386

[30] Lopez L Colan SD Frommelt PC Ensing GJ Kendall K Younoszai AK Recommendations for quantification methods during the performance of a pediatric echocardiogram: a report from the pediatric measurements writing group of the American society of echocardiography pediatric and congenital heart disease council. *J Am Soc Echocardiogr.* (2010) 23:465–95; quiz 576–7. 10.1016/j.echo.2010.03.019 20451803

[31] Wu L Dong B Liu X Hong W Chen L Gao K Standard echocardiographic view recognition in diagnosis of congenital heart defects in children using deep learning based on knowledge distillation. *Front Pediatr.* (2022) 9:770182. 10.3389/fped.2021.770182 35118028 PMC8805220

[32] Passalis N Tzelepi M Tefas A. Probabilistic knowledge transfer for lightweight deep representation learning. *IEEE Trans Neural Netw Learn Syst.* (2021) 32:2030–9. 10.1109/TNNLS.2020.2995884 32479404

[33] He K Zhang X Ren S Sun J. Deep residual learning for image recognition. In: *Proceedings of the 2016 IEEE Conference on Computer Vision and Pattern Recognition (CVPR).* Las Vegas, NV (2016). p. 770–8. 10.1109/CVPR.2016.90

[34] Zhang H Wu C Zhang Z Zhu Y Zhang Z Lin H Resnest: split-attention networks. *arXiv* [Preprint]. (2020). arXiv:2004.08955. 10.3390/s21134612 34283160 PMC8272209

[35] Ronneberger O Philipp F Thomas B. U-net: convolutional networks for biomedical image segmentation. In: *Proceedings of the International Conference on Medical Image Computing and Computer-Assisted Intervention (MICCAI).* Munich (2015). p. 234–41. 10.1007/978-3-319-24574-4_28

[36] Huang G Liu Z Van Der Maaten L Weinberger K. Densely connected convolutional networks. In: *Proceedings of the 2017 IEEE Conference on Computer Vision and Pattern Recognition (CVPR).* Honolulu, HI (2017). p. 4700–8. 10.1109/CVPR.2017.243

[37] Fu J Liu J Jiang J Li Y Bao Y Lu H. Scene segmentation with dual relation-aware attention network. *IEEE Trans Neural Netw Learn Syst.* (2021) 32:2547–60. 10.1109/TNNLS.2020.3006524 32745005

[38] Tian Z Shen C Chen H He T. FCOS: a simple and strong anchor-free object detector. *IEEE Trans Pattern Anal Mach Intell.* (2020) 19:1922–33. 10.1109/TPAMI.2020.3032166 33074804

[39] Lin T Dollár P Girshick R He K Hariharan B Belongie S. Feature pyramid networks for object detection. In: *Proceedings of the 2017 IEEE Conference on Computer Vision and Pattern Recognition (CVPR).* Honolulu, HI (2017). p. 936–44. 10.1109/CVPR.2017.106

